# Endodontic Treatment of a Maxillary Lateral Incisor with Two Canals: A Case Report

**DOI:** 10.22037/iej.v13i3.20986

**Published:** 2018

**Authors:** Eshaghali Saberi, Shima Bijari, Forough Farahi

**Affiliations:** a *Department of Endodontics, Oral and Dental Diseases Research Center, Dental School, Zahedan University of Medical Sciences, Zahedan, Iran*

**Keywords:** Anatomic Variation, Root Canal, Tooth Abnormalities

## Abstract

Variations in the number of roots and canals have been extensively reported in endodontic literature. One rare variation is presence of two separate root canals in maxillary lateral incisors. This study reports a maxillary lateral incisor with two canals. Although rare, knowledge about this anatomical variation can help in successful endodontic treatment of such teeth.

## Introduction

One important step in root canal treatment is to find, clean and disinfect all root canals. Knowledge about the variations of the root canal system of all teeth directly affects the outcome of endodontic treatment [1]. Presence of multiple canals in maxillary lateral incisors is a rare occurrence. According to Green [2], Pineda and Kuttler [3] and Vertucci [4], maxillary incisors are single-canal in 100% of the cases, and presence of multiple canals in these teeth is limited to case reports available in the literature [5-15]. Due to such a low prevalence rate, additional canals in maxillary incisors may be missed by dentists. On the other hand, the shape of crown is often normal in such teeth, and the additional canals are sometimes detected by routine radiographic examination [8]. 

This study reports endodontic treatment of a maxillary lateral incisor with two separate canals.

## Case Report

**Figure 1:**
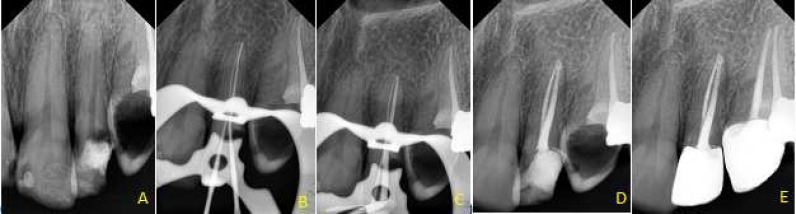
*A)* Preoperative periapical radiography of the maxillary left lateral incisor; *B)* radiography taken after detecting the root canals; *C)* Radiography taken for evaluation of master cone; *D)* Periapical radiography taken after completion of root canal filling; *E)* Follow-up radiography

A 35-year-old female was referred to Dental School of Zahedan University of Medial Sciences by her general dentist for consultation about endodontic treatment of her maxillary left lateral incisor (tooth #10). She complained of continuous vague pain in her anterior maxilla. Her medical history was unremarkable. Her maxillary left lateral incisor already had a prepared access cavity and did not respond to the vitality tests. It did not have pain on palpation or percussion. Her oral hygiene was moderate and she did not have any periodontal pocket around her maxillary left lateral incisor. The tooth did not have any sinus tract and the patient had no history of dental trauma. Radiographic examination revealed an unusual root canal system. The lake of centralization of the main canal. Non-surgical root canal treatment was planned for her. Local anesthesia was administered by supra-periosteal injection of 2% lidocaine plus 1:80000 epinephrine (Persocaine, DarouPakhsh, Tehran, Iran). Temporary dressing was removed and the access cavity outline was corrected to find possible anatomical variations. A rubber dam was placed for isolation of the tooth. One orifice was evident in the pulp chamber floor. The orifice of the second canal was found following negotiating the pulp chamber floor with a #10 K-file (Dentsply Maillefer, Ballaigues, Switzerland) and precurving the file palatally. Another radiograph was taken, which confirmed the presence of a second canal (Figure 1). 

The canals underwent mechanical and chemical debridement with M-two rotary files (25/0.06, 20/0.06, 15/0.05, 10/0.04) (VDW GmbH, Germany) for the palatal canal and up to 35/0.04, for the buccal canal using the standard technique. Irrigation was performed using 5.25% sodium hypochlorite. 

After root canal instrumentation, 17% EDTA (Asia Chimi Teb, Tehran, Iran) was used to eliminate the smear layer. After rinsing with saline, the canals were dried with sterile paper points and filled with gutta-percha (Meta Biomed Co., Chungcheongbuk do, Korea) and AH-26 sealer (Dentsply, DeTrey, Konstanz, Germany) using lateral compaction technique. The tooth was then temporarily restored (Figure 1D). The patient was referred to prosthetic specialist for permanent restoration and a follow-up radiography was taken after six months (Figure1E). 

## Discussion

Anatomical variations and dental anomalies such as fusion, gemination, dens in dent, palatogingival groove, distolingual groove and some changes in normal development of the Hertwig’s epithelial root sheath may be suggested for maxillary incisors with two canals or two roots [16-19]. According to a previous study, the prevalence of multiple canals is 3% in maxillary incisors [20]. 

Tooth gemination is a type of dental anomaly characterized by division of tooth during its development resulting in formation of two crowns with one root. In dental fusion, two separate tooth buds are fused during crown development resulting in formation of a tooth with double crowns and two canals in one root [18, 21]. 

In our case, preoperative clinical and radiographic examinations confirmed normal size of the crown. Thus, fusion (one large crown) and gemination (fused crowns) were ruled out. 

Reports regarding lateral incisors with dens in dent or dens invagination and two roots are scarce [15, 22-27]. In our case, preoperative clinical examination did not reveal invagination of enamel or dentin that could cause dens in dent. Other developmental anomalies such as palatogingival groove or distolingual groove were also ruled out by clinical examination. 

According to Bhashkar [18], normal root development occurs when the Hertwig’s epithelial root sheath is bended horizontally at the cementoenamel junction to narrow the cervical opening. In our reported case, the canals had a buccopalatal position. Except for two case reports [28, 29], most of the root canals of such teeth are positioned mesiodistally [17, 30-32]. 

Precise radiographic interpretation and taking radiographs from different angles as well as comprehensive knowledge about the morphology and variations of the root canal system aid the clinician in better detection of such cases. In most cases, use of magnifying loupes or a microscope can also be helpful. Moreover, novel radiographic modalities such as cone-beam computed tomography, which is a high-resolution three-dimensional technique can greatly help in detection of such anatomical variations [33]. Cone-beam computed tomography was not used for our patient since there was no doubt regarding the presence of a second canal, and other anomalies were all ruled out.

## Conclusion

Our reported case highlights the significance of having comprehensive knowledge about the anatomy and morphology of root canals to reach a correct diagnosis. Radiographic examination from different angles and clinical examination are required for all teeth.
